# Supplementary material for: Convolutional neural network for automated mass segmentation in mammography
Source: BMC Bioinformatics. 2020 Dec 9;21(Suppl 1):192. doi: 10.1186/s12859-020-3521-y (PMC7724817; doi:10.1186/s12859-020-3521-y)
Supplement: Supplementary file 1 — Additional file 1 Supplementary materials (semantic segmentation using FCN, semantic segmentation using SegNet, semantic segmentation using Dilated-Net, localization using Faster R-CNN, comparison between state-of-the-art DL models, Supplementary Table 1, Supplementary Figures 1–4). [file 12859_2020_3521_MOESM1_ESM.pdf]

Supplementary materials

Pre-processing

The AMF [1] is a nonlinear filter that removes impulse noise while preserving edges and corners to improve the image quality. The CLAHE filter increases the contrast between the masses and their surrounding tissues [2–5]. The CLAHE [1] filter operates on small regions in the image, called tiles, rather than the entire image. It calculates the contrast for each tile individually producing local histograms. Each tile's contrast is enhanced and the neighboring tiles are then combined using bilinear interpolation to eliminate artificially induced boundaries. The contrast in the homogeneous regions can be limited using a clipLimit factor to avoid amplifying any noise that might be present in the image. We used tiles of [8 8] and a clipLimit factor of 0.005 with the CLAHE technique. Figure 1 shows a sample of the combined data-set we used in our experiments. Figure 2 shows images containing suspicious areas and its associated pixel-level GTMs. All full MGs and GTMs are converted into png format and re-sized to 512×512. All pixels in the GTM are labeled as belonging to the background (0) or breast lesion (255) (see Fig. 2).

Semantic segmentation using FCN

Semantic segmentation is an active research area for medical images where deep CNNs are used to classify each pixel in the image individually. Semantic segmentation results in a map image that is segmented by classes. The fully convolutional network (FCN) [6] is an encoder-decoder network. The encoder path uses a pre-trained VGG16 model [7] and transfer their learned representations by fine-tuning to the segmentation task. The decoder path uses up-sampling operations, and replace the final fully connected layer (FCL) with an N×1×1 convolution layer, which output probabilities for N classes. A skip architecture is proposed by [6] where the weights of shallow, fine layer features are combined with deep, coarse layer features to produce accurate and detailed segmentations, as intensive up-sampling can lead to coarse segmentation maps. There are 3 versions of FCN (FCN-32s, FCN-16s, FCN-8s) based on VGG16 network [6]. In this research, we adapt the FCN-8s VGG16 based network [6] to our segmentation task. FCN-8s up-samples the final feature map by a factor of 8 after fusing feature maps from the third and fourth max-pooling layers.

Semantic segmentation using SegNet

The SegNet architecture [8] adopts the VGG16 network [7] along with an encoder-decoder framework wherein it drops the FCLs of the network. SegNet shares a similar architecture to the encoder-decoder U-Net described in the previous subsection. However, in SegNet, the indices at each max-pooling layer in the encoder contracting path at each level are stored and later used to up-sample the corresponding feature map in the decoder by unpooling it using those stored indices (Fig. 3). Storing the indices from the contraction path helps keep the high-frequency information intact, however, it also misses neighboring information when unpooling from low-resolution feature maps. Finally, a Softmax classifier is used to produce the final segmentation maps with the same resolution of the original MG image. In this work, we used a Segnet that is pre-initialized with layers and weights from a pre-trained VGG16 model with an encoder D of 5.

Semantic segmentation using Dilated-Net

Recently, Dilated-Net [9], also known as atrous convolutions, have been used in different image segmentation tasks [10–14]. Dilated convolutions [9] allow us to explicitly control the resolution at which feature responses are computed and incorporate larger context without increasing the number of parameters or the amount of computation. We adopt the dilated CNN in [9] with some modification to the network. The implemented dilated CNN architecture consists of ten cascaded 3×3 convolutional layers with dilation factors 1, 1, 2, 4, 8, 16, 32, 1, 1 and 1 (Fig. 4). Figure 4 illustrates a 3×3 convolution kernels with different dilation factor as 1,2, and 3. The last three layers are FCLs of 1×1 convolutions followed by dropout of 0.5 [15]. The first nine convolutional layers are followed by BN layer [16] and a ReLU activation function [17]. To classify the pixels, the last convolutional layer has two 1×1 convolutions, followed by a Softmax classifier.

Localization using Faster R-CNN

We adapt the Faster R-CNN method proposed in [18] to compare its performance in terms of accuracy of detection and inference time with that of the proposed Vanilla U-Net model. Faster R-CNN is based on a VGG16 model [7] with additional components for detecting, localizing and classifying lesions in MG image. Faster R-CNN outputs a BB for each detected lesion, and a score, which reflects the confidence in the class of the lesion. The Faster R-CNN method in [18] is trained with our pre-processed, and augmented data-set. Further details about the implemented Faster R-CNN method can be found in the original article in [18]. One limitation stated in the study of [18] is that the training data-set comes from small sized publicly available pixel-level annotated data-set. However, in our study we are using our combined large-sized data-set to reproduce their work.

Semantic segmentation using region growing (RG)

We also implemented the region growing (RG) model proposed in [19] and apply it to our MG images. RG is a traditional image segmentation CAD model that starts with selecting an initial seed point and then groups pixels or sub-regions into larger regions according to a similarity criterion. As RG results are sensitive to the initial seeds, the automated accurate seed selection is very critical for image segmentation. Further details about the implemented RG method can be found in the original article [19].

Comparison between state-of-the-art DL methods

Table 1 lists the information about the architecture, databases, the number of images, the evaluation methods (i.e. Accuracy (ACC.), area under curve (AUC), Dice index (DI)), TPR@FPR, the testing time per image as provided in the literature.

Author details

References

1. Zuiderveld K. Contrast limited adaptive histogram equalization. Graphics gems. 1994;p. 474–485.
2. Dhungel N, Carneiro G, Bradley AP. Deep learning and structured prediction for the segmentation of mass in mammograms. In: International Conference on Medical Image Computing and Computer-Assisted Intervention. Springer; 2015. p. 605–612.
3. Dhungel N, Carneiro G, Bradley AP. A deep learning approach for the analysis of masses in mammograms with minimal user intervention. Medical image analysis. 2017;37:114–128.
4. Al-antari MA, Al-masni MA, Choi MT, Han SM, Kim TS. A fully integrated computer-aided diagnosis system for digital X-ray mammograms via deep learning detection, segmentation, and classification. International Journal of Medical Informatics. 2018;117:44–54.
5. Carneiro G, Nascimento J, Bradley AP. Automated Analysis of Unregistered Multi-View Mammograms With Deep Learning. IEEE transactions on medical imaging. 2017;36(11):2355–2365.
6. Long J, Shelhamer E, Darrell T. Fully convolutional networks for semantic segmentation. In: Proceedings of the IEEE conference on computer vision and pattern recognition; 2015. p. 3431–3440.
7. Simonyan K, Zisserman A. Very deep convolutional networks for large-scale image recognition. arXiv preprint arXiv:14091556. 2014;.
8. Badrinarayanan V, Kendall A, Cipolla R. Segnet: A deep convolutional encoder-decoder architecture for image segmentation. arXiv preprint arXiv:151100561. 2015;.
9. Yu F, Koltun V. Multi-scale context aggregation by dilated convolutions. arXiv preprint arXiv:151107122. 2015;.
10. Chen LC, Papandreou G, Kokkinos I, Murphy K, Yuille AL. Deeplab: Semantic image segmentation with deep convolutional nets, atrous convolution, and fully connected crfs. IEEE transactions on pattern analysis and machine intelligence. 2018;40(4):834–848.
11. Noothout JM, de Vos BD, Wolterink JM, Išgum I. Automatic segmentation of thoracic aorta segments in low-dose chest CT. In: Medical Imaging 2018: Image Processing. vol. 10574. International Society for Optics and Photonics; 2018. p. 105741S.
12. Yu F, Koltun V, Funkhouser TA. Dilated Residual Networks. In: CVPR. vol. 2; 2017. p. 3.
13. Vo DM, Lee SW. Semantic image segmentation using fully convolutional neural networks with multi-scale images and multi-scale dilated convolutions. Multimedia Tools and Applications. 2018;p. 1–19.
14. Wolterink JM, Leiner T, Viergever MA, Išgum I. Dilated convolutional neural networks for cardiovascular MR segmentation in congenital heart disease. In: Reconstruction, Segmentation, and Analysis of Medical Images. Springer; 2016. p. 95–102.
15. Srivastava N, Hinton G, Krizhevsky A, Sutskever I, Salakhutdinov R. Dropout: a simple way to prevent neural networks from overfitting. The Journal of Machine Learning Research. 2014;15(1):1929–1958.
16. Ioffe S, Szegedy C. Batch normalization: Accelerating deep network training by reducing internal covariate shift. arXiv preprint arXiv:150203167. 2015;.
17. Nair V, Hinton GE. Rectified linear units improve restricted boltzmann machines. In: Proceedings of the 27th international conference on machine learning (ICML-10); 2010. p. 807–814.
18. Ribli D, Horváth A, Unger Z, Pollner P, Csabai I. Detecting and classifying lesions in mammograms with Deep Learning. Scientific reports. 2018;8(1):4165.
19. Melouah A. Comparison of automatic seed generation methods for breast tumor detection using region growing technique. In: IFIP International Conference on Computer Science and its Applications. Springer; 2015. p. 119–128.
20. Choukroun Y, Bakalo R, Ben-Ari R, Akselrod-Ballin A, Barkan E, Kisilev P. Mammogram Classification and Abnormality Detection from Nonlocal Labels using Deep Multiple Instance Neural Network. 2017;.
21. Akselrod-Ballin A, Karlinsky L, Alpert S, Hasoul S, Ben-Ari R, Barkan E. A region based convolutional network for tumor detection and classification in breast mammography. In: Deep Learning and Data Labeling for Medical Applications. Springer; 2016. p. 197–205.
22. Akselrod-Ballin A, Karlinsky L, Hazan A, Bakalo R, Horesh AB, Shoshan Y, et al. Deep Learning for Automatic Detection of Abnormal Findings in Breast Mammography. In: Deep Learning in Medical Image Analysis and Multimodal Learning for Clinical Decision Support. Springer; 2017. p. 321–329.
23. Dhungel N, Carneiro G, Bradley AP. The automated learning of deep features for breast mass classification from mammograms. In: International Conference on Medical Image Computing and Computer-Assisted Intervention. Springer; 2016. p. 106–114.
24. Platania R, Shams S, Yang S, Zhang J, Lee K, Park SJ. Automated breast cancer diagnosis using deep learning and region of interest detection (bc-droid). In: Proceedings of the 8th ACM International Conference on Bioinformatics, Computational Biology, and Health Informatics. ACM; 2017. p. 536–543.
25. Teuwen J, van de Leemput S, Gubern-Mérida A, Rodriguez-Ruiz A, Mann R, Bejnordi BE. Soft tissue lesion detection in mammography using deep neural networks for object detection. 2018;.
26. Al-masni MA, Al-antari MA, Park J, Gi G, Kim TY, Rivera P, et al. Detection and classification of the breast abnormalities in digital mammograms via regional convolutional neural network. In: 2017 39th Annual International Conference of the IEEE Engineering in Medicine and Biology Society (EMBC). IEEE; 2017. p. 1230–1233.
27. Al-masni MA, Al-antari MA, Park JM, Gi G, Kim TY, Rivera P, et al. Simultaneous detection and classification of breast masses in digital mammograms via a deep learning YOLO-based CAD system. Computer methods and programs in biomedicine. 2018;157:85–94.
28. Sun W, Tseng TLB, Zheng B, Qian W. A preliminary study on breast cancer risk analysis using deep neural network. In: International Workshop on Digital Mammography. Springer; 2016. p. 385–391.
29. Reiazi R, Paydar R, Ardakani AA, Etedadialiabadi M. MAMMOGRAPHY LESION DETECTION USING FASTER R-CNN DETECTOR. Computer Science & Information Technology;p. 111.

List of tables

Table 1 Shows a comparison between the proposed segmentation method and the current state-of-the-art DL methods for segmentation or localization of lesions in MG images.

List of figures

Fig. 1 The databases used in our experiments.
Fig. 2 MG images and their corresponding GTMs.
Fig. 3 In Segnet, the indices at each max-pooling layer in the encoder contracting path at each level are stored and later used to up-sample the corresponding feature map in the decoder by unpooling it using those stored indices.
Fig. 4 Architecture of the dilated-Net, containing ten convolutional layers with dilation factors, indicated in red, increasing from 1 in the first layer to 32 in the seventh layer. The last 1×1 convolutional layer is followed by a Softmax classifier.

Table 1

Paper	Approach	Tested on	#Images	Size	TL	Aug.	AUC	ACC.	DI	TPR@FPR	Inference time/image
**Proposed method**	Vanilla U-Net	INbreast,<br>DDSM,<br>UCHCDM	17,140	512×512	Yes	Yes	-	0.926	0.951	-	0.12 sec
**Rilbi et al. [18]**	Faster R-CNN	INbreast	3,927	1400×1700	Yes	Yes	95.0	N/A	N/A	0.90@0.3	N/A
**Choukroun et al. [20]**	Batch-based CNN	INbreast<br>INbreast	410<br>2,500	224×224<br>224×224	Yes<br>Yes	Yes<br>Yes	72.2<br>83.1	N/A<br>N/A	N/A<br>N/A	N/A<br>N/A	N/A<br>N/A
**Akselrod et al. [21]**	Faster R-CNN	In-house	850	800×800	Yes	Yes	72.0	77.0	N/A	0.93@0.56	0.2 sec
**Akselrod et al. [22]**	Faster R-CNN	INbreast<br>INbreast	100<br>310	224×224<br>224×224	Yes<br>Yes	Yes<br>Yes	N/A<br>N/A	N/A<br>N/A	N/A<br>N/A	0.93@0.56<br>0.8@1.5	5 sec<br>5 sec
**Dhungel et al. [23]**	R-CNN+<br>Random forest (RF)	INbreast	410	40×40	Yes	Yes	N/A	95.0	N/A	0.91@1.3	41 sec
**Dhungel et al. [3]**	R-CNN+ RF	INbreast	410	40×40	Yes	Yes	91.0	95.0	85	0.9@1.0	41 sec
**Platania et al. [24]**	YOLO	DDSM	10,480	128×128	Yes	Yes	N/A	90.0	N/A	N/A	1.25 sec
**Teuwen et al. [25]**	Faster R-CNN<br>Mask R-CNN	In-house<br>In-house	23,405<br>23,405	227×227<br>227×227	Yes<br>Yes	Yes<br>Yes	N/A<br>N/A	N/A<br>N/A	N/A<br>N/A	0.87@0.56<br>0.97@3.5	N/A<br>N/A
**Al-masni et al. [26]**	YOLO	DDSM	600	448×448	No	No	87.74	96.33	N/A	0.96@2.6	N/A
**Al-masni et al. [27]**	YOLO	DDSM	600	448×448	No	Yes	96.45	99.70	N/A	N/A	3 sec
**Sun et al. [28]**	Batch-based CNN	In-house	840	52×52	No	Yes	69.82	N/A	N/A	N/A	N/A
**Reiazi et al. [29]**	Faster R-CNN	MIAS	120	75×75	No	No	N/A	N/A	N/A	N/A	N/A

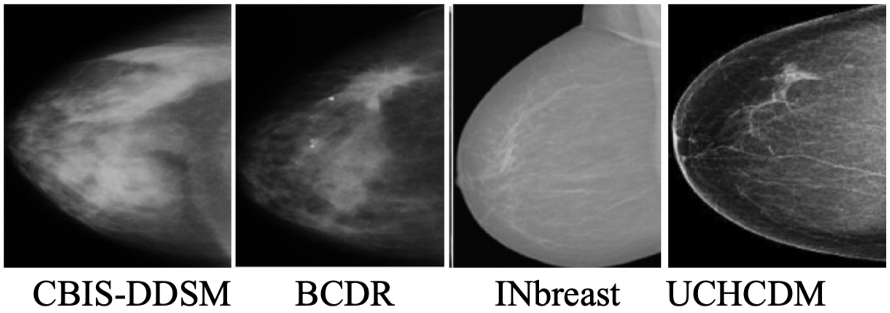

Fig. 1

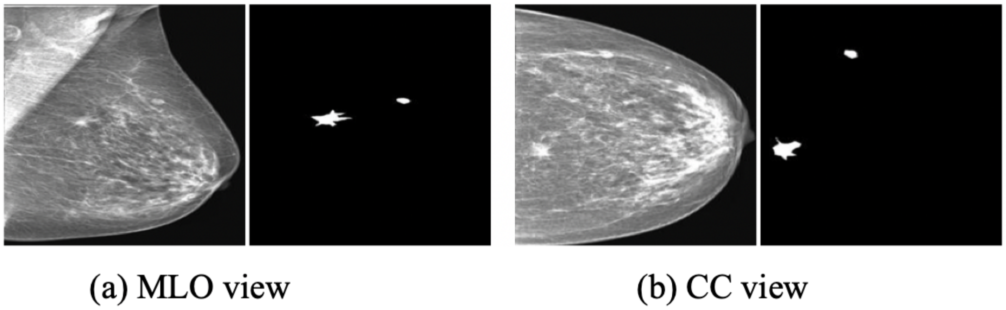
(a) MLO view
(b) CC view

Fig. 2

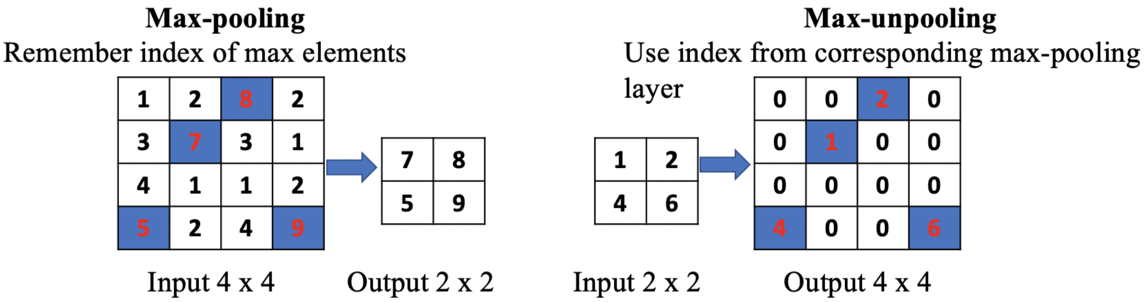
Max-pooling
Remember index of max elements
Input 4 x 4
Output 2 x 2
Max-unpooling
Use index from corresponding max-pooling layer
Input 2 x 2
Output 4 x 4

Fig. 3

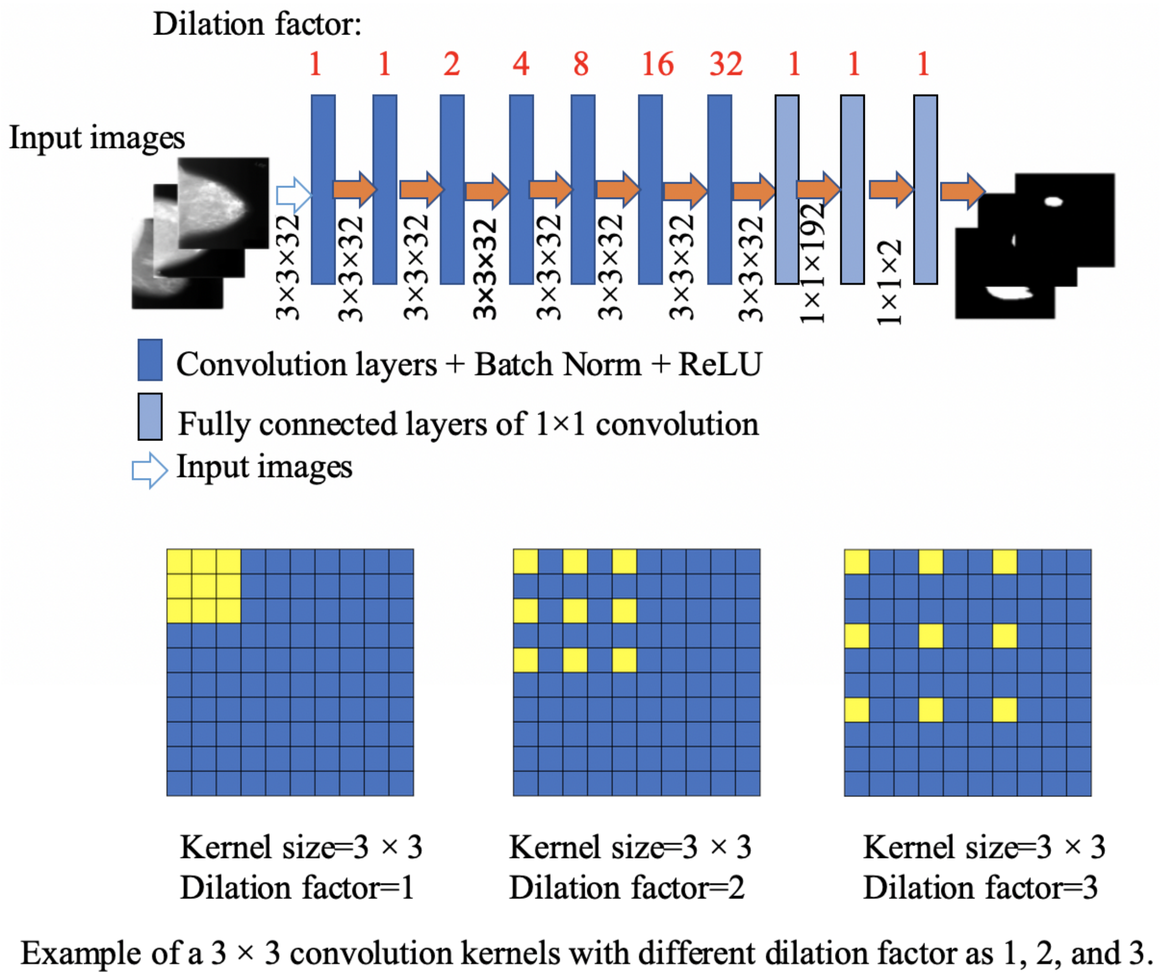
Dilation factor:
1 1 2 4 8 16 32 1 1 1
Input images
3×3×32
3×3×32
3×3×32
3×3×32
3×3×32
3×3×32
3×3×32
3×3×32
1×1×192
1×1×2
Convolution layers + Batch Norm + ReLU
Fully connected layers of 1×1 convolution
Input images
Kernel size=3 × 3
Dilation factor=1
Kernel size=3 × 3
Dilation factor=2
Kernel size=3 × 3
Dilation factor=3
Example of a 3 × 3 convolution kernels with different dilation factor as 1, 2, and 3.

Fig. 4
